# The risk of acute infection in association with first ever diagnosed depression: a cohort study

**DOI:** 10.1007/s00127-024-02784-1

**Published:** 2024-11-09

**Authors:** Noah Aebi, Christoph R. Meier, Susan S. Jick, Undine Lang, Julia Spoendlin

**Affiliations:** 1 Basel Pharmacoepidemiology Unit, Division of Clinical Pharmacy and Epidemiology, Department of Pharmaceutical Sciences, University of Basel, Basel, Switzerland; 2 Hospital Pharmacy, University Hospital Basel, Basel, Switzerland; 3 University Psychiatric Clinics Basel, University Hospital Basel, Basel, Switzerland; 4 Boston Collaborative Drug Surveillance Program, Lexington, MA USA; 5 Boston University School of Public Health, Boston, MA USA

**Keywords:** Depression, infection risk, acid Sphingomyelinase, CPRD

## Abstract

**Purpose:**

To assess the risk of acute infections in patients with first ever diagnosed depression compared to patients with no diagnosed depression in a primary-care database.

**Methods:**

We conducted a cohort study using the UK CPRD GOLD database (2000–2019). We identified patients aged 18 years or older with a recorded Read code for depression (cohort entry date) and compared them to patients with no Read codes for depression using risk set sampling. Comparison groups were frequency-matched on age and sex, and comparison patients were required to have ≥ 1 general practitioner (GP) contact within 14 days before cohort entry. The primary outcome was a composite of outpatient diagnosed acute infections, including respiratory, gastrointestinal, urogenital infections and septicemia) within the two-years after cohort entry. We applied propensity score fine stratification and estimated incidence rates and IR ratios (IRR) using negative binomial regression.

**Results:**

In a weighted population of 285,922 patients with diagnosed depression and 285,921 comparison patients, the IR of acute infections was 97.3/1000 person-years (py) in patients with and 83.7/1000 py in patients with no diagnosed depression. The weighted IRR of acute infection was 1.18 (95% CI 1.16–1.20) comparing those with and with no diagnosed depression. Excluding patients with baseline comorbidities yielded an IRR even closer to the null: 1.07 (95% CI, 1.04–1.09).

**Conclusions:**

Our results suggest that patients with diagnosed depression are not at a meaningfully increased risk of acute infections compared to patients with no diagnosed depression. Slightly increased overall relative risks of infections can be explained by residual differences in health care utilization and by the severity of comorbidities.

**Supplementary Information:**

The online version contains supplementary material available at 10.1007/s00127-024-02784-1.

## Introduction

The lifetime prevalence of major depressive episodes is estimated to be between 15% and 18% worldwide [1–4]. 

In vitro studies have indicated that alterations in the immune system are present in patients with depression [5–7]. However, the extent to which these changes result in an increased risk of acute infections in the general population is poorly understood [8, 9].

Two previous observational studies reported an up to 60% increased risk of infection in patients with depression compared to patients without depression [10, 11]. One study was conducted using Danish health registry data, which are representative of the overall Danish population (1995–2012). The second study was a survey among US college students (2004). However, neither study controlled for important potential confounders such as BMI, comorbidities, or health care utilization (surveillance bias). Furthermore, the most frequently recorded infectious disease in the Danish study was hepatic infection (even before respiratory infections) [11–13], which is likely not representative of the infection burden of the overall general population. Thus, the robustness and representativeness of these prior results is questionable. Overall, evidence of the association between depression and the risk of infection in the general population is scarce.

We conducted an observational cohort study using a UK-based primary care database to compare the risk of acute infections between adults with and with no diagnosed depression.

## Patients and methods

### Study design and data source

We conducted a cohort study using data from the UK Clinical Practice Research Datalink (CPRD) GOLD, a primary care database that contains de-identified health data on some 14 million patients recorded by general practitioners (GPs) [14–16]. CPRD GOLD has been described extensively elsewhere [14–16] but briefly, it captures information on patient demographics, lifestyle factors, diagnoses, medication prescriptions, laboratory test results, and referrals to secondary care.

### Study population

We conducted a cohort study comparing patients with an incident diagnosis of depression between January 1, 2000, and December 31, 2019, defined as having a first ever recorded Read code for depression (Read-code list available in supplement) to patients with no recorded Read code for depression. All patients were aged 18 years or older.

All patients were required to have at least 2 years of recorded history in CPRD GOLD before the cohort entry date (date of first depression diagnosis). Patients who had a prescription for an antidepressant at any time before the cohort entry date were excluded. If a code for suicidal ideation or suicide attempt was recorded within one year prior to the first diagnosis of depression, the cohort entry date was set to the first record of suicidal ideation (*N* = 3984, 0.6% of all patients with diagnosed depression) [17]. For each exposed patient, we selected one comparison patient with the same cohort entry date, but without a recorded Read code for depression at any time prior to the cohort entry date of the corresponding exposed patients. Comparison patients were matched to exposed patients on year of birth, sex, and post code and were required to have at least one GP visit within the last 14 days before cohort entry date to ensure that the patient was actively enrolled with his or her GP at that time.

We further excluded patients with a recorded diagnosis of alcoholism or HIV at any time before the cohort entry date, as well as those with a diagnosis of cancer / ongoing chemotherapy or with use of immunosuppressive drugs within two years before the cohort entry date.

### Study outcomes

The primary outcome was a composite of four types of infections identified using Read diagnosis codes for (1) acute respiratory, (2) genitourinary, or (3) gastrointestinal infections, as well as (4) septicaemia. All recorded infections during follow-up were captured; thus, a patient could contribute more than one outcome event. A time lag of at least 14 days was required between two records of infection of the same type to be counted as separate events. Code lists are available in the supplement. In a secondary analysis, we evaluated each type of infection separately, not censoring for occurrence of another type of infection.

Additionally, we identified all patients in the study population with HES linkage (approx. 50%). In this subset, we identified hospital-diagnosed infections (composite outcome of the same four types of acute infections included in the main analyses) using ICD-10 codes (ICD-10 codes are listed in the supplement).

### Follow-up

We accumulated person time for patients with and with no diagnosed depression (exposed or non-exposed) starting on the first day after the cohort entry date until censoring. Patients were censored on the date the first of the following occurred: they became a case, an exclusion criterion was recoded, death, end of data recording, the end of the study period, or after a maximum of 630 days (2 years) of follow up. Patients with diagnosed depression were censored after 12 months (on day 366) if there was no further record for either a prescription of an antidepressant or a repeated diagnosis of depression. In addition, we censored comparison patients when a diagnosis of depression or a prescription for an antidepressant was recorded during follow-up.

### Patient characteristics and covariables

We evaluated 32 baseline covariables, including demographics, lifestyle factors, and comorbidities associated with health care utilization at any time prior to the cohort entry date. We further assessed a history of cancer > 2 years prior to the cohort entry date, or a recorded diagnosis for pneumonia within 180 days prior to cohort entry date. We further captured prescriptions for immunosuppressive medication recorded within 180 days prior to the cohort entry date, as well as the number of GP contacts (health care utilization) within 1 year before the cohort entry date. All covariables are listed in Table 1.

**Table 1:** Characteristics of patients with and without first ever diagnosed depression at cohort entry before and after PS-weighting

	Before PS-weighting No. (%)		After PS-weighting No. (%)	
	**Diagnosed** **depression**	**Without diagnosed depressio**n	**Diagnosed depression**	**Without diagnosed depressio**n
**No. of patients**	285 923	285 923	285 922	285 921
**Age**,** mean (SD)**,** y**	38.9 (17.5)	38.9 (17.5)	38.9 (17.5)	39.3 (17.5)
**Women**	177 438 (62.1)	177 438 (62.1)	177 438 (62.1)	178 089 (62.3)
**No. of GP visits (365 d prior)**,** mean (SD)**	8.3 (8.4)	9.4 (8.7)	8.3 (8.4)	8.5 (7.8)
**BMI**				
12.0-18.4	7 668 (2.7)	6 896 (2.4)	7 668 (2.7)	7 627 (2.7)
18.5–24.9	93 362 (32.7)	97 825 (34.2)	93 362 (32.7)	92 318 (32.3)
25.0–29.9	62 780 (22)	66 741 (23.3)	62 780 (22)	63 310 (22.1)
30.0–60.0	47 443 (16.6)	47 173 (16.5)	47 443 (16.6)	48 642 (17)
Unknown	74 670 (26.1)	67 288 (23.5)	74 669 (26.1)	74 024 (25.9)
**Smoking status**				
Non-smoker	120 950 (42.3)	153 100 (53.5)	120 949 (42.3)	118 675 (41.5)
Current smoker	76,753 (26.8)	53,181 (18.6)	76 753 (26.8)	78 659 (27.5)
Past smoker	55 152 (19.3)	54 536 (19.1)	55 152 (19.3)	55 429 (19.4)
Unknown	33 068 (11.6)	25 106 (8.8)	33 068 (11.6)	33 158 (11.6)
**Alcohol consuption**				
Low risk consumption ( < = 14 units/week)	187 612 (65.6)	193 630 (67.7)	187 612 (65.6)	188 315 (65.9)
High risk consumption (14 + units/week)	15 481 (5.4)	14 458 (5.1)	15 481 (5.4)	15 497 (5.4)
Unknown	82 830 (29)	77 835 (27.2)	82 829 (29)	82 110 (28.7)
**Comedication (180 d prior)**				
Opioid	13 936 (4.9)	13 854 (4.8)	13 936 (4.9)	14 880 (5.2)
Antipsychotics	6 266 (2.2)	6 304 (2.2)	6 266 (2.2)	6 574 (2.3)
Agents acting on RAS	19 927 (7)	27 795 (9.7)	19 927 (7)	21 464 (7.5)
Beta-blocker	16 314 (5.7)	19 233 (6.7)	16 314 (5.7)	17 395 (6.1)
Calcium channel blockers	11 850 (4.1)	16 011 (5.6)	11 850 (4.1)	12 852 (4.5)
Diuretics	17 621 (6.2)	22 960 (8)	17 621 (6.2)	19 167 (6.7)
Statins	18 596 (6.5)	23 838 (8.3)	18 596 (6.5)	20 245 (7.1)
Thrombocyte aggregation inhibitors	15 547 (5.4)	17 514 (6.1)	15 547 (5.4)	16 782 (5.9)
Anticoagulants	2 802 (1)	4 607 (1.6)	2 802 (1)	3 271 (1.1)
Proton pump inhibitors	21 359 (7.5)	24 683 (8.6)	21 359 (7.5)	22 531 (7.9)
Systemic corticosteroids	8 939 (3.1)	12 031 (4.2)	8 939 (3.1)	9 504 (3.3)
Immunosuppressant	502 (0.2)	896 (0.3)	502 (0.2)	599 (0.2)
**Comorbidities**				
Asthma	51 150 (17.9)	56 603 (19.8)	51 150 (17.9)	50 958 (17.8)
COPD	3 907 (1.4)	3 432 (1.2)	3 907 (1.4)	4 098 (1.4)
Arrhythmia	4 404 (1.5)	4 791 (1.7)	4 404 (1.5)	4 574 (1.6)
Congestive heart failure	2 857 (1)	2 858 (1)	2 857 (1)	3 026 (1.1)
Ischemic heart disease	9 149 (3.2)	9 084 (3.2)	9 149 (3.2)	9 763 (3.4)
Myocardial infarction	4 132 (1.4)	4 357 (1.5)	4 132 (1.4)	4 464 (1.6)
Hyperlipidaemia	13 268 (4.6)	15 598 (5.5)	13 268 (4.6)	13 985 (4.9)
Diabetes mellitus	12 026 (4.2)	17 710 (6.2)	12 025 (4.2)	13 141 (4.6)
DVT / PE	833 (0.3)	1 027 (0.4)	833 (0.3)	866 (0.3)
Ulcer	5 004 (1.8)	4 430 (1.5)	5 004 (1.8)	5 162 (1.8)
Diagnosed renal diseases	5 970 (2.1)	6 289 (2.2)	5 970 (2.1)	6 146 (2.1)
Hypothyroidism	9 874 (3.5)	13 796 (4.8)	9 874 (3.5)	10 455 (3.7)
History of cancer (non-active, last code ≥ 730 days before CED)	7 580 (2.7)	8 227 (2.9)	7 580 (2.7)	7 818 (2.7)
Pneumonia (any time up to 180 days prior)	5 907 (2.1)	5 726 (2)	5 907 (2.1)	5 947 (2.1)
**C-statistic**	0.60		0.50	

Abbreviations: BMI, body mass index; CED, Cohort entry date; COPD, chronic obstructive pulmonary disease; DVT, deep vein thrombosis, GP, general practitioner; PE, pulmonary embolism, RAS, renin-angiotensin- system; SD, standard deviation; Std Diff, absolute standardised difference;

### Statistical analyses

To control for confounding, we applied inverse probability weighting by fine strata of the propensity score (PS) [18]. We calculated a PS based on all 32 baseline covariables (Table 1). We formed 50 PS strata based on the PS distribution in the cohort of diagnosed depression and weighted the cohort of patients with no diagnosed depression proportionally to the distribution of the exposed cohort in the PS stratum in which it fell. We trimmed the non-overlapping portions of the PS distributions to exclude non-comparable patients. The absolute standardized differences before and after weighting were used to assess balance of covariables. To obtain the measure of overall balance of covariables, we quantified the crude and weighted C-statistic (ideal balance c = 0.5) [19]. We assessed the distribution of baseline covariables for patients with diagnosed depression and patients with no diagnosed depression before and after PS weighting. We calculated crude and PS-weighted incidence rates (IRs) with 95% confidence intervals (CIs) for the two different cohorts separately. In addition, we quantified crude and PS-weighted IR ratios (IRRs) with 95% CIs for the four different types of infections by comparing patients with diagnosed depression to patients with not diagnosed depression. We calculated IRRs using negative binomial regression [20]. Whenever the negative binomial model did not converge, we used Poisson regression corrected for over dispersion [21].

### Subgroup and sensitivity analyses

We performed analyses in subgroups of sex and age (18–24, 25–34, 35–44, 45–54, 55–64, and 65 + years).

To evaluate potential confounding by health care utilization (surveillance bias), we conducted subgroup analyses stratified by number of GP contacts within one year before cohort entry date (0–2, 3–5, 6–9,10–19, and 20 + GP visits). In an additional analysis, we removed the requirement for patients without a depression diagnosis to have GP contact within 14 days before cohort entry date. We performed this additional analysis to evaluate the possible influence of surveillance bias.

Depression may be a secondary consequence of chronic illness [22, 23]. Many chronic illnesses are also known risk factors for acute infections [24–26]. To evaluate potential confounding by underlying chronic illnesses, we performed a sensitivity analysis in which we excluded all patients with a record for any of the 14 baseline comorbidities within one year prior to cohort entry date, or with one or more prescriptions for any of 12 comedications within 180 days prior to the cohort entry date.

To validate the robustness of our outcomes, we performed two additional analyses with varying minimum time periods between separate episodes of infections of 7 and 28 days.

To assess whether a history of cancer and/or immunosuppression more than 2 years prior to cohort entry date is associated with susceptibility to acute infections, we performed a sensitivity analysis in which we excluded all patients who had a history of cancer and/or were prescribed an immunosuppressant at any time before cohort entry date (patients with a record for cancer or immunosuppression < = 2 years prior to the cohort entry date were already excluded).

To evaluate the potential role of severity of depression, we conducted an additional analysis in which we sub-divided the exposed group into two subgroups, treated or untreated depression, and compared them each to patients with no diagnosed depression. We censored follow-up for treated depression 180 days after the last antidepressant prescription. We stopped follow-up 180 days after the last depression diagnosis in patients with untreated depression.

Among patients with HES linkage, we calculated the proportion of patients with hospitalized infections in the exposed and non-exposed comparison groups separately. Additionally, we compared baseline characteristics between patients with or with no diagnosed depression who had a hospital-diagnosed infection.

All analyses were performed using SAS 9.4 software (SAS Institute, Cary, NC).

## Results

**Fig. 1:**
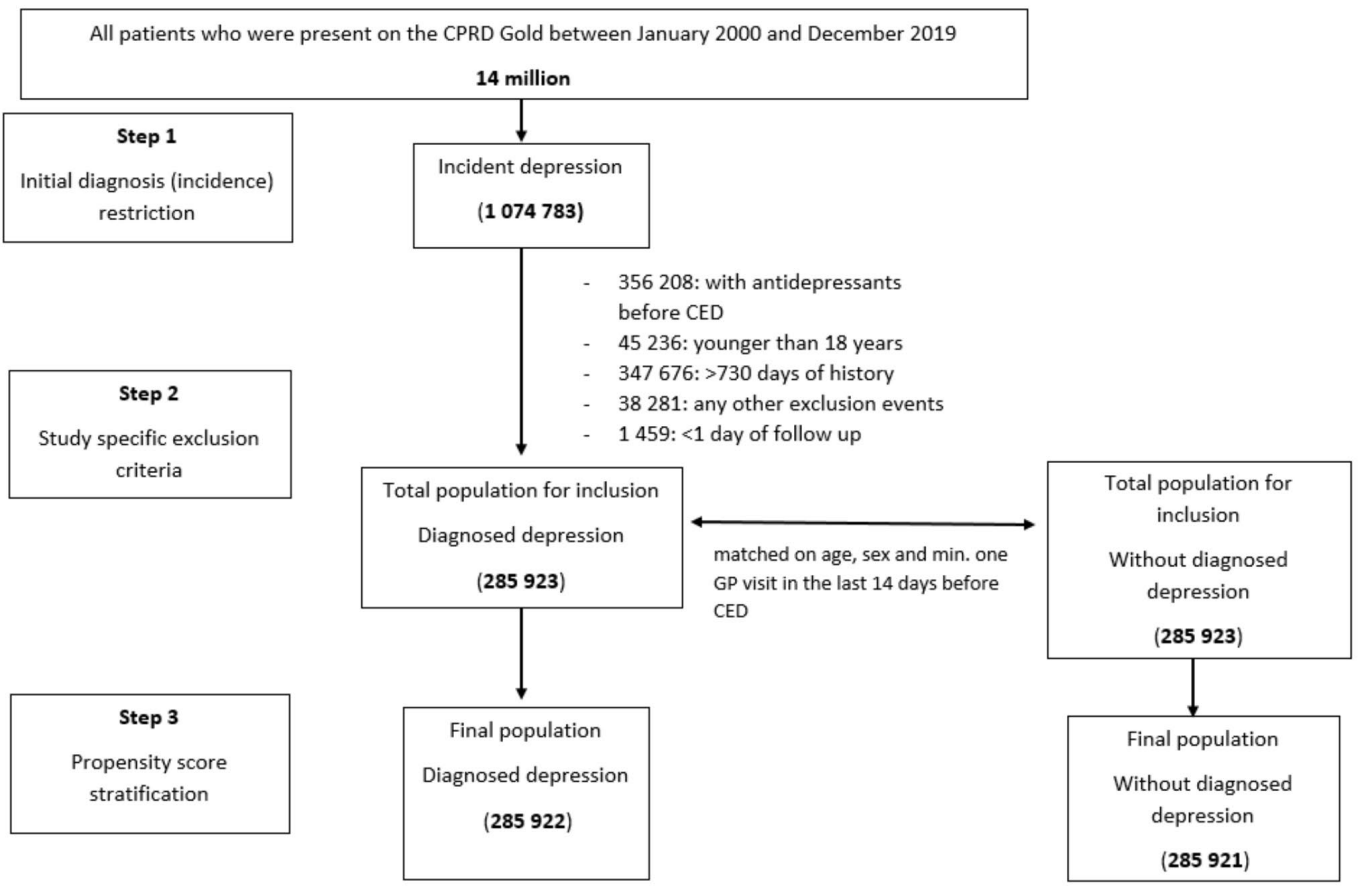
Flowchart of study population

After weighting, we included 285 922 patients with first ever diagnosed depression who met our study criteria and matched 285 921 patients with no diagnosed depression. The mean age at cohort entry was 38.9 years (SD; 17.5) for patients with diagnosed depression and 39.3 years (SD; 17.5) in the comparison group, and 62.1% were female. In total, 66.6% of patients with diagnosed depression received a prescription for an antidepressant on the same day as the depression diagnosis (Flow Chart in Fig. 1). All baseline covariables were well balanced after PS-weighting (weighted c-statistic = 0.50, Table 1).

The crude and PS-weighted IRs and IRRs for the primary and secondary outcomes are shown in Table 2. In the results, we focused on the weighted outcomes. Crude results are displayed in Tables 2 and 3, and S1; overall they did not differ materially from PS-weighted results.

**Table 2:** Incidence Rates (95% CI) and Incidence Rates Ratios (95% CI) of diagnosed acute respiratory, genitourinary, gastrointestinal infections and septicaemia among patients with and without first ever diagnosed depression

	No. of Events	Total person-years of follow-up	Incidence rate per 1000 person-years (95% CI)	IRR (95% CI)
**Any acute infection**				
**No diagnosed depression**				
Crude	42 349	486 551.1	87.0 (86.2–87.9)	ref
PS-weighted	40 840	488 121.5	83.7 (82.9–84.5)	
**Diagnosed depression**				
Crude	29 949	292 940.0	102.2 (101.1–103.4)	1.17 (1.16–1.19)
PS-weighted	29 949	292 939.0	102.2 (101.1–103.4)	1.19 (1.17–1.21)
**Acute respiratory infections**				
**No diagnosed depression**				
Crude	26 389	486 551.1	54.2 (53.6–54.9)	ref
PS-weighted	25 468	488 121.5	52.2 (51.5–52.8)	
**Diagnosed depression**				
Crude	18 175	292 940.0	62.0 (61.1–63.0)	1.14 (1.12–1.17)
PS-weighted	18 175	292 939.0	62.0 (61.1–63.0)	1.15 (1.13–1.18)
**Acute genitourinary infections**				
**No diagnosed depression**				
Crude	14 976	486 551.1	30.8 (30.3–31.3)	ref
PS-weighted	14 439	488 121.5	29.6 (29.1–30.1)	
**Diagnosed depression**				
Crude	11 011	292 940.0	37.6 (36.9–38.3)	1.22 (1.18–1.26)
PS-weighted	11 011	292 939.0	35.6 (35.0–36.3)	1.24 (1.20–1.28)
**Acute gastrointestinal infections**				
**No diagnosed depression**				
Crude	863	486 551.1	1.8 (1.7–1.9)	ref
PS-weighted	823	488 121.5	1.7 (1.6–1.8)	
**Diagnosed depression**				
Crude	681	292 940.0	2.3 (2.2–2.5)	1.31 (1.18–1.45)
PS-weighted	681	292 939.0	2.2 (2.0–2.4)	1.31 (1.19–1.45)
**Acute septicaemia**				
**No diagnosed depression**				
Crude	239	486 551.1	0.5 (0.4–0.6)	ref
PS-weighted	224	488 121.5	0.5 (0.4–0.5)	
**Diagnosed depression**				
Crude	175	292 940.0	0.6 (0.5–0.7)	1.22 (0.99–1.49)
PS-weighted	175	292 939.0	0.6 (0.5–0.7)	1.23 (1.00–1.50)

Abbreviations: CI, confidence interval; IR, incidence rate; IRR incidence rate ratio; PS, propensity score;

**Table 3:** IRR for acute infections comparing patients with first ever diagnosed depression to patients with no diagnosed depression restricted to patients without any recorded study comorbidities

Variable	IRR (95% CI)	
	Crude	PS-weighted
**Without recorded comorbidities or comedications**		
Any acute infection	1.06 (1.03–1.09)	1.09 (1.07–1.12)
Acute respiratory infections	1.07 (1.04–1.10)	1.10 (1.06–1.13)
Acute genitourinary infections	1.04 (0.99–1.08)	1.07 (1.03–1.12)
Acute gastrointestinal infections	1.20 (1.03–1.40)	1.20 (1.04–1.40)
Acute septicaemia	1.15 (0.79–1.67)	1.15 (0.79–1.67)

After PS-weighting, 29 949 acute infections (primary outcome) were recorded during 292 939 person-years (py) of follow-up for patients with first ever diagnosed depression, resulting in an IR of 97.3 acute infections/1000 py. In the comparison cohort of patients with no diagnosed depression, 40 840 acute infections were recorded during 488 121.5 py, yielding an IR of 83.7 /1000 py. The weighted IRR for all acute infections comparing patients with first ever diagnosed depression to patients with no diagnosed depression was 1.19 (95% CI, 1.17–1.21) (Table 2). Exclusion of patients with a history of cancer (IRR 1.19, 95% CI, 1.17–1.21), or lengthening (IRR 1.19, 95% CI, 1.19–1.21) or shortening (IRR 1.19, 95% CI, 1.17–1.21) the minimum time period between separate outcome events of infection did not meaningfully change our results. (Table S1)

**Fig. 2:**
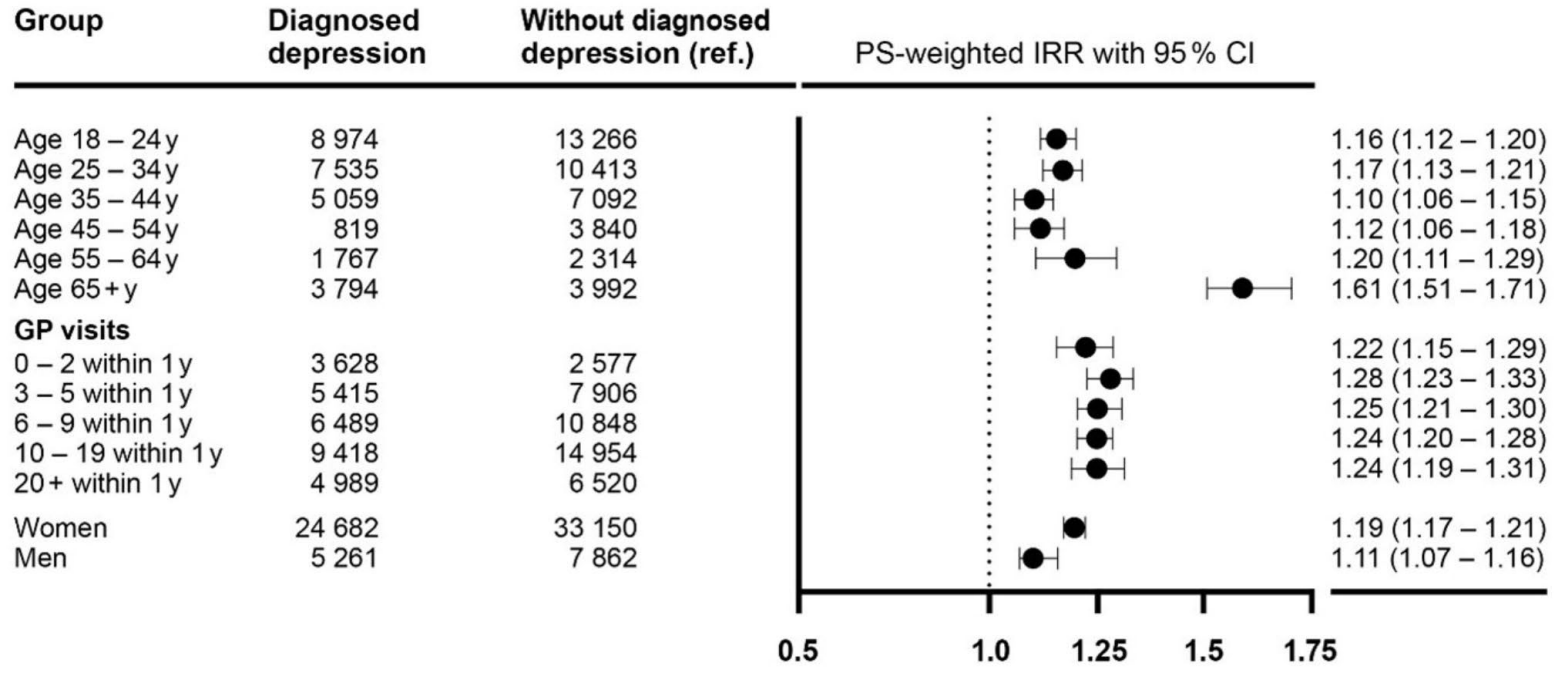
Result of subgroup analyses for the risk of any acute infection comparing patients with first ever diagnosed depression to patients with no diagnosed depression after PS-weighting

Detailed results of the subgroup and sensitivity analyses can be found in Tables 3 and 4; Fig. 2, and in Supplementary Tables S1-S6.

**Table 4:** PS-weighted IRR for acute infections in patients with untreated or treated diagnosed depression compared with patients with no diagnosed depression

	Untreated diagnosed depression vs. no diagnosed depression	Treated diagnosed depression vs. no diagnosed depression
	**PS Weighted IRR (95% CI)**	
**Any acute infection**	1.03 (1.00–1.06)	1.25 (1.22–1.27)
**Acute respiratory infections**	1.03 (0.99–1.06)	1.21 (1.18–1.24)
**Acute genitourinary infections**	0.75 (0.73–0.77)*	1.30 (1.25–1.34)
**Acute gastrointestinal infections**	1.14 (0.96–1.36)	1.43 (1.27–1.60)
**Acute septicaemia**	1.27 (0.91–1.76)	1.14 (0.89–1.44)

Abbreviations: CI, confidence interval; IR, incidence rate; IRR, incidence rate ratios; PS, propensity score; *, POISSON

Incidence rates for the composite outcome of acute infection were highest (123.1/1000py) in patients under 25 years of age and lowest in patients aged 55–65 years. Despite the high absolute risk of infections in patients under 25 years of age, the weighted IRR was not materially increased (1.15, 95% CI, 1.12–1.19), whereas the weighted IRR for any acute infection was highest (1.50, 95% CI, 1.40–1.60) in the subgroup of patients aged 65 years and older. (Fig. 2)

The IR of new diagnosed infection was three times higher in women with first ever diagnosed depression (IR 127.6/1000py) compared to men (IR 46.9/1000py). (Fig. 2) This increase was mainly accounted for by genitourinary infections (IR 50.2/1000py in women and 11.2/1000py in men). However, IRRs for the composite outcome were not meaningfully different between women (IRR 1.18, 95% CI, 1.16–1.21) and men (IRR 1.10, 95% Cl, 1.06–1.15). (Fig. 2)

The IRRs of acute infections were similar across all strata of number of GP visits (Fig. 2).

In the additional analysis, in which the requirement to have a recorded GP visit at least once in the 14 days before cohort entry date was removed for patients without a depression diagnosis, the IRR for the primary outcome was significantly increased in patients with diagnosed depression compared to patients with no diagnosed depression (crude IRR, 1.60; 95% CI, 1.59–1.61, PS-weighted IRR 1.54; 95% CI, 1.52–1.55).

When we restricted the analysis to patients with no recorded diagnoses of any of the 14 baseline comorbidities at any time prior to the cohort entry date, and no prescriptions for any of 12 comedications within 180 days before cohort entry date, the IRR for any acute infection in patients with first ever diagnosed depression compared to no diagnosed depression was almost null: weighted IRR 1.09 (95% CI, 1.07–1.12) (Table 3). The same pattern was seen for all four primary outcomes when evaluated separately (Table 3).

The PS-weighted IRR for any acute infection in treated depression compared to no diagnosed depression was 1.25 (95% CI, 1.22–1.27). When we compared patients with untreated diagnosed depression to patients with no diagnosed depression, the PS-weighted IRR was 1.03 (95% CI, 1.00-1.06) for the composite outcome of any infection (Table 4).

The number of patients in our study population with a hospital-diagnosed infection was too small to yield interpretable results.

## Discussion

In this large retrospective cohort study, there was no meaningful difference in risk of infection between patients with first ever diagnosed depression compared to patients with no diagnosed depression after controlling for confounding and surveillance bias. Excluding patients with a history of one or more recorded comorbidity or comedication before cohort entry further attenuated the initially observed minimal excess risk of acute infection (IRR 1.09). Thus, our results suggest that the risk of infection in association with diagnosed depression is lower than previously reported. The difference in results may be explained by control for underlying comorbidities and surveillance bias among patients with secondary depression.

The IRR of 1.19 in this study is lower than the IRR of 1.61 (95% CI, 1.49–1.74) reported in a previous national Danish registry-based cohort study [11]. The Danish study evaluated infections diagnosed in inpatient and outpatient settings. Results were adjusted for age and sex, but not for health care utilization or any other potential confounders. As not all patients seek medical attention for infection, patients who are in closer contact with the health care system (e.g. patients with diagnosed depression) are more likely to have an infection diagnosed than patients who do not see their GP regularly, potentially introducing surveillance bias. We controlled for the number of GP contacts within a year before cohort entry and required exposed and comparison patients to have at least one recorded GP visit within 14 days prior to cohort entry. In the analysis in which we dropped the latter requirement for non-exposed patients, the adjusted IRR increased to 1.54 for acute infection in patients with diagnosed depression compared to no diagnosed depression. This risk estimate is similar to the result in the Danish study, suggesting that their results may be explained by differential health care utilization. Prior studies have shown, that the lack of control for health care utilization between comparison groups may introduce bias [27]. 

This hypothesis is further supported by the fact that relative risks moved closer to the null after restricting to patients with no recorded study comorbidities or comedications. After this restriction we observed an IRR for any infection of interest of 1.09 (95% CI 1.07–1.12) and for genitourinary infections of 1.07 (95% CI, 1.03–1.12. (Table 3). Unlike common acute respiratory and gastrointestinal infections, urinary tract infections (UTIs) commonly require antibiotic treatment and are thus likely to be more completely reported to the GP. Thus, surveillance bias can be expected to play less of a role when analyzing this outcome compared to the other infections in this study.

A previous retrospective study analyzed survey data from the 2004 American College Health Association-National College Health Assessment (ACHA-NCHA) and reported a higher risk of any infection, with ORs ranging from 1.10 for ear infection to 1.30 for bronchitis among college students with self-reported depression compared to college students without self-reported depression. However, only a few potential confounding factors were controlled for in this survey study. Furthermore, college students who all lived on campus were substantially different than the adult primary-care population in this study, limiting comparability [10]. In addition, survey studies may be affected by selection (volunteer) bias compared to population bases studies such as this one.

Patients with first ever diagnosed depression may differ from patients with no diagnosed depression in many ways. Therefore, inferring a causal association between diagnosed depression and infection must be done with caution. To explore the role of potential confounding factors, we conducted several subgroup and sensitivity analyses. To evaluate the role of underlying comorbidities, which may have led to secondary depression, we excluded all patients with a recorded diagnosis for any of the 14 evaluated pre-existing comorbidities or for a co-medication within 180 days prior to the cohort entry date. Among the 55% of remaining patients with potentially primary diagnosed depression, the excess relative risk of developing an acute infection dropped from 19 to 9% compared to no diagnosed depression, suggesting that the severity of underlying comorbidities may have introduced residual confounding and that the risk of acute infection may be at least partially mediated by the underlying health status of patient with diagnosed depression.

To evaluate the role of the severity of diagnosed depression, we categorized patients with first ever diagnosed depression into those with or without a recorded prescription for an antidepressant on the day of first diagnosis. We assumed that patients receiving an antidepressant suffer from more severe depression. When comparing patients without an antidepressant, we no longer observed an increased risk of acute infection after PS-weighting (IRR, 1.03; 95% CI, 1.00-1.06). In contrast, the risk of acute infection was minimally increased for patients with diagnosed depression and pharmacological antidepressant therapy (PS-weighted IRR, 1.25; 95% CI, 1.22–1.27). After excluding patients with underlying comorbidities, the IRR decreased to 1.09 (95% CI, 1.107–1.12), suggesting that the severity of underlying pre-existing diseases may play a role in this minimal increase in risk.

We found a threefold increased absolute risk of acute infection for women compared to men. This difference is accounted for primarily by genitourinary infections. Genitourinary infections are much more common in women, and since most UTIs require antibiotic treatment, they are often recorded by GPs. However, the relative risk of acute infections overall was not meaningfully different between men and women in our study population.

Patients with diagnosed depression aged 65 years or older had an IRR of 1.5 for acute infection compared to patients with no diagnosed depression in the same age group. However, 88% of patients with diagnosed depression in this age group had at least one recorded pre-existing comorbidity, which may have confounded the result. When we excluded patients 65 or older with pre-existing comorbidities, the IRR of any acute infection decreased to 1.30 (95% CI of 1.02–1.67).

### Strengths and limitations

Our cohort study was based on a large and representative study population and used rigorous methodology including several sensitivity and subgroup-analyses to comprehensively assess the role of confounding and bias. PS-fine stratification allowed us to adjust for recorded differences between cohorts.

However, some limitations need to be considered. First, GP-recorded IRs especially of acute respiratory and gastrointestinal infections do not reflect the true IR of such infections in the general UK population. Mild infections are generally not recorded if patients do not seek medical advice, which is common for infections such as acute respiratory and gastrointestinal infections, which often do not require GP-prescribed pharmacological treatment. Genitourinary infections and septicaemia are likely more completely entered in the GP record because they are more severe and typically require antibiotic therapy [28–30]. Severe infections (i.e. septicaemia and hospital recorded infections) were sparse and sample size was too small to be informative. Thus, our results address only diagnosed depression and moderate infections, which led to a contact with the GP but were not severe. Secondly, this study did not encompass all types of infections. We focused on four types of relatively frequently diagnosed infections that and are typically encountered in GP practices. We were also not able to differentiate between viral and bacterial infections, as this information is not routinely recorded in CPRD GOLD data. Thirdly, it is likely that some patients who have depression were undiagnosed. We can therefore not rule out some exposure misclassification where patients with undiagnosed depression are included in the control group. If the presence of depression does increase the risk of infection, this would have biased our results towards the null [31]. Moreover, receiving a diagnosis of depression per se may be associated with other factors that protect from infection when compared to patients with undiagnosed depression, which we could not control for. Fourth, it is possible that the cause for GP consultation in the unexposed cohort may have been associated with an increased risk of infection (e.g. diabetes, COPD) in a proportion of patients with no diagnosed depression. However, this likely did not meaningfully confound our results given that IRRs were closer to the null after excluding all patients with recorded comorbidities. We required comparison patients to have at least 1 GP visit within 2 weeks before CED, because prior studies have shown that omission to control for health care utilization between comparison groups introduces surveillance bias [27]. However, residual confounding cannot be completely ruled out.

## Conclusions

This study suggests that patients with diagnosed depression are not at a meaningfully increased risk for the here examined acute infections when compared to patients with no diagnosed depression after adjusting for surveillance bias and comorbidities. Lack of control for health care seeking behaviour may explain the increased risks of infection in association with diagnosed depression seen in previous studies.

## Electronic Supplementary Material

Below is the link to the electronic supplementary material.

Supplementary Material 1

## Data Availability

No datasets were generated or analysed during the current study.

## Abbreviations

CED: Cohort entry date
CPRD: Clinical Practice Research Datalink
HIV: Human immunodeficiency virus
PS: Propensity score
