# Supplementary material for: The risk of acute infection in association with first ever diagnosed depression: a cohort study
Source: Soc Psychiatry Psychiatr Epidemiol. 2024 Nov 9;60(7):1761–70. doi: 10.1007/s00127-024-02784-1 (PMC12238197; doi:10.1007/s00127-024-02784-1)
Supplement: Supplementary file 1 — Supplementary Material 1 [file 127_2024_2784_MOESM1_ESM.docx]

Supplement

**Code lists**

**Depression (ICD-10 F32-33)**

;Depressive episode (F32)

depressive episode 18171

[X]Depressive episode 34028

[X]Single episode of depressive reaction 2419

[X]Single episode of psychogenic depression 112

[X]Single episode of reactive depression 1823

;Mild depressive episode (F32.0)

depressive episode, mild 3220

depression

depressive episode 7452

[X]Major depression; mild

;Moderate depressive episode (F32.1)

depressive episode, moderate 1471

[X]Moderate depressive episode 8757

[X]Major depression moderately severe 463

;Severe depressive episode without psychotic symptoms (F32.2)

depressive episode, severe, without psychosis 132

Agitated depression 40409

Agitated depression 24359

[X]Single episode agitated depressn w'out psychotic symptoms 27

[X]Single episode major depression w'out psychotic symptoms 166

[X]Single episode vital depression w'out psychotic symptoms 1

[X]Severe depressive episode without psychotic symptoms 928

[X]Major depression; severe without psychotic symptoms

[X]Vital depression, recurrent without psychotic symptoms

;Severe depressive episode with psychotic symptoms (F32.3)

depressive episode, severe, with psychosis 61

[X]Severe depressive episode with psychotic symptoms 428

[X]Single episode of major depression and psychotic symptoms 50

[X]Single episode of psychogenic depressive psychosis 9

[X]Single episode of psychotic depression 127

[X]Single episode of reactive depressive psychosis 25

[X]Major depression; severe with psychotic symptoms

;Other depressive episodes (F32.8)

depression 1641

[X]Single episode of masked depression NOS

[X]Other depressive episodes 195

[X]Atypical depression 76

;Depressive episode, unspecified (F32.9)

depressive episode NOS 933

depressive episode, unspecified 242

[X]Depressive episode, unspecified 16418

[X]Depression NOS 211985

[X]Depressive disorder NOS 4868

;Recurrent depressive disorder (F33)

Recurrent major depressive episode 8939

Recurrent depression 13690

Seasonal affective disorder 625

[X]Reactive depression NOS 23983

[X]Recurrent depressive disorder 2806

[X]Recurrent episodes of depressive reaction 594

[X]Recurrent episodes of psychogenic depression 167

[X]Recurrent episodes of reactive depression 1146

[X]Seasonal depressive disorder 26

Seasonal affective disorder 1364

;Recurrent depressive disorder, current episode mild (F33.0)

Recurrent major depressive episodes, mild 523

[X]Recurrent depressive disorder, current episode mild 130

;Recurrent depressive diosrder, current episode moderate (F33.1)

Recurrent major depressive episodes, moderate 2990

[X]Recurrent depressive disorder, current episode moderate 387

;Recurrent depressive disorder, current episode severe without psychotic symptoms (F33.2)

Recurrent major depressive episodes, severe, no psychosis 142

[X]Recurr depress disorder cur epi severe without psyc sympt 56

[X]Major depression, recurrent without psychotic symptoms 78

;Recurrent depressive disorder, current episode severe with psychotic symptoms (F33.3)

Recurrent major depressive episodes, severe, with psychosis 108

Depressive psychoses 3657

depressive psychosis 617

Psychotic reactive depression 1110

[X]Recurrent depress disorder cur epi severe with psyc symp 32

[X]Recurr severe episodes/major depression+psychotic symptom 49

[X]Recurr severe episodes/psychogenic depressive psychosis 12

[X]Recurrent severe episodes of psychotic depression 89

[X]Recurrent severe episodes/reactive depressive psychosis 19

;recurrent depressive disorder, currently in remission (F33.4)

Recurrent major depressive episodes, in full remission 406

Recurrent major depressive episodes,partial/unspec remission 189

[X]Recurrent depressive disorder, currently in remission 174

;Other recurrent depressive disorders (F33.8)

[X]Other recurrent depressive disorders 8

;Recurrent depressive disorder, uspecified (F33.9)

Recurrent major depressive episode NOS 193

[X]Monopolar depression NOS 7

Recurrent major depressive episodes, unspecified 70

[X]Recurrent depressive disorder, unspecified 18

;general

Depressive disorder NEC 389945

depression 14710

depressive episode, partial or unspec remission 272

depressive episode, in full remission 102

Endogenous depression first episode 4667 (thought as genetical predisposition to suffer from depression)

Endogenous depression first episode 35826

Endogenous depression 42074

Endogenous depression - recurrent 42002

Atypical depressive disorder 129

Drug-induced depressive state

[X]Endogenous depression without psychotic symptoms 158

[X]Endogenous depression with psychotic symptoms 132

[X]Prolonged single episode of reactive depression 131

Depressive personality disorder

;unclear

Symptoms of depression

Depressive symptoms

;1JJ..00 Suspected depression

[X]Antenatal depression

Neurotic depression reactive type

[X]Vital depression; recurrent without psychotic symptoms

[X]Depressive conduct disorder

;F530.0: Mild mental and behavioural disorders associated with the puerperium, not elsewhere classified

[X]Postnatal depression NOS

[X]Postpartum depression NOS

Postnatal depression

**Respiratory infections (J01 - J06, J09 - J12, J20.3 - J20.7, J21.0 - J21.1)**

;acute sinusitis (J01)

H01..00 Acute sinusitis

H01..11 Sinusitis

H010.00 Acute maxillary sinusitis

H011.00 Acute frontal sinusitis

H012.00 Acute ethmoidal sinusitis

H013.00 Acute sphenoidal sinusitis

H014.00 Acute rhinosinusitis

H01y.00 Other acute sinusitis

H01y000 Acute pansinusitis

H01yz00 Other acute sinusitis NOS

H01z.00 Acute sinusitis NOS

H130.12 Maxillary sinusitis

H131.11 Frontal sinusitis

H13y100 Pansinusitis

Hyu0000 [X]Other acute sinusitis

;acute pharyngitis (J02)

A340200 Streptococcal pharyngitis

AA12.00 Vincent's pharyngitis

AA25.11 Rhinopharyngitis mutilans

H00..00 Acute nasopharyngitis

H02..00 Acute pharyngitis

H02..13 Throat infection - pharyngitis

H020.00 Acute gangrenous pharyngitis

H021.00 Acute phlegmonous pharyngitis

H022.00 Acute ulcerative pharyngitis

H023.00 Acute bacterial pharyngitis

H023000 Acute pneumococcal pharyngitis

H023100 Acute staphylococcal pharyngitis

H023z00 Acute bacterial pharyngitis NOS

H024.00 Acute viral pharyngitis

H02z.00 Acute pharyngitis NOS

H050.00 Acute laryngopharyngitis

H271100 Influenza with pharyngitis

Hyu0100 [X]Acute pharyngitis due to other specified organisms

;acute tonsillitis (J03)

H03..00 Acute tonsillitis

H03..11 Throat infection - tonsillitis

H03..12 Tonsillitis

H030.00 Acute erythematous tonsillitis

H031.00 Acute follicular tonsillitis

H032.00 Acute ulcerative tonsillitis

H033.00 Acute catarrhal tonsillitis

H034.00 Acute gangrenous tonsillitis

H035.00 Acute bacterial tonsillitis

H035000 Acute pneumococcal tonsillitis

H035100 Acute staphylococcal tonsillitis

H035z00 Acute bacterial tonsillitis NOS

H036.00 Acute viral tonsillitis

H037.00 Recurrent acute tonsillitis

H03z.00 Acute tonsillitis NOS

Hyu0200 [X]Acute tonsillitis due to other specified organisms

;acute laryngitis and tracheitis (J04)

A340100 Streptococcal laryngitis

AA1z.11 Vincent's laryngitis

H04..00 Acute laryngitis and tracheitis

H040.00 Acute laryngitis

H040000 Acute oedematous laryngitis

H040100 Acute ulcerative laryngitis

H040200 Acute catarrhal laryngitis

H040300 Acute phlegmonous laryngitis

H040400 Acute haemophilus influenzae laryngitis

H040500 Acute pneumococcal laryngitis

H040600 Acute suppurative laryngitis

H040w00 Acute viral laryngitis unspecified

H040x00 Acute bacterial laryngitis unspecified

H040z00 Acute laryngitis NOS

H04z.00 Acute laryngitis and tracheitis NOS

H055.00 Pharyngolaryngitis

H160400 Laryngitis sicca

H271000 Influenza with laryngitis

; Acute obstructive laryngitis [croup] and epiglottitis (J05)

H043200 Acute obstructive laryngitis

H043211 Croup

H044.00 Croup

H043.00 Acute epiglottitis (non strep)

H043000 Acute epiglottitis without obstruction

H043100 Acute epiglottitis with obstruction

H043z00 Acute epiglottitis NOS

; Acute upper respiratory infections of multiple and unspecified sites (J06)

H050.00 Acute laryngopharyngitis

H05..00 Other acute upper respiratory infections

Hyu0.00 [X]Acute upper respiratory infections

Hyu0300 [X]Other acute upper respiratory infections/multiple sites

; Influenza due to certain identified influenza viruses (J09)

1J72.00 Suspected influenza A virus subtype H1N1 infection

1W0..00 Possible influenza A virus H1N1 subtype

H2A..00 Influenza due to Influenza A virus subtype H1N1

H2A..11 Influenza A (H1N1) swine flu

; Influenza due to other identified influenza virus (J10)

H040400 Acute haemophilus influenzae laryngitis

H060800 Acute haemophilus influenzae bronchitis

H060C00 Acute bronchitis due to parainfluenza virus

H2...00 Pneumonia and influenza

H202.00 Pneumonia due to parainfluenza virus

H222.00 Pneumonia due to haemophilus influenzae

H222.11 Pneumonia due to haemophilus influenzae

H27..00 Influenza

H270.00 Influenza with pneumonia

H270.11 Chest infection - influenza with pneumonia

H270000 Influenza with bronchopneumonia

H270100 Influenza with pneumonia; influenza virus identified

H270z00 Influenza with pneumonia NOS

H271.00 Influenza with other respiratory manifestation

H271000 Influenza with laryngitis

H271100 Influenza with pharyngitis

H271z00 Influenza with respiratory manifestations NOS

H27y.00 Influenza with other manifestations

H27y000 Influenza with encephalopathy

H27y100 Influenza with gastrointestinal tract involvement

H27yz00 Influenza with other manifestations NOS

H27z.00 Influenza NOS

H27z.12 Influenza like illness

H29..00 Avian influenza

H2y..00 Other specified pneumonia or influenza

H2z..00 Pneumonia or influenza NOS

Hyu0500 [X]Influenza+other manifestations;influenza virus identified

; Influenza due to unidentified influenza virus (J11)

Hyu0600 [X]Influenza+oth respiratory manifestatns;virus not identifd

Hyu0700 [X]Influenza+other manifestations; virus not identified

; Viral pneumonia, not elsewhere classified (J12)

H20..00 Viral pneumonia

H20..11 Chest infection - viral pneumonia

H20y.00 Viral pneumonia NEC

H20z.00 Viral pneumonia NOS

H200.00 Pneumonia due to adenovirus

H201.00 Pneumonia due to respiratory syncytial virus

H202.00 Pneumonia due to parainfluenza virus

H203.00 Pneumonia due to human metapneumovirus

H20y.00 Viral pneumonia NEC

H20z.00 Viral pneumonia NOS

H21..11 Chest infection - pneumococcal pneumonia

Hyu0800 [X]Other viral pneumonia

H2...00 Pneumonia and influenza

H25..00 Bronchopneumonia due to unspecified organism

H25..11 Chest infection - unspecified bronchopneumonia

;Acute bronchitis due to Mycoplasma pneumoniae (J20.1)

H060A00 Acute bronchitis due to mycoplasma pneumoniae

;Acute bronchitis due to coxsackievirus(J20.3)

H060B00 Acute bronchitis due to coxsackievirus

;Acute bronchitis due to parainfluenza virus (J20.4)

H060C00 Acute bronchitis due to parainfluenza virus

;Acute bronchitis due to respiratory syncytial virus (J20.5)

H060D00 Acute bronchitis due to respiratory syncytial virus

;Acute bronchitis due to rhinovirus (J20.6)

H060E00 Acute bronchitis due to rhinovirus

;Acute bronchitis due to echovirus

H060F00 Acute bronchitis due to echovirus

;Acute bronchitis, unspecified (J20.9)

H06..00 Acute bronchitis and bronchiolitis

H060v00 Subacute bronchitis unspecified

H060w00 Acute viral bronchitis unspecified

H060x00 Acute bacterial bronchitis unspecified

H060z00 Acute bronchitis NOS

H06z.00 Acute bronchitis or bronchiolitis NOS

H30..00 Bronchitis unspecified

Hyu1000 [X]Acute bronchitis due to other specified organisms

; Acute broncholitis (J21)

H061.00 Acute bronchiolitis

H061200 Acute bronchiolitis with bronchospasm

H061z00 Acute bronchiolitis NOS

; Acute bronchiolitis due to respiratory syncytial virus (J21.0)

H061500 Acute bronchiolitis due to respiratory syncytial virus

;Acute bronchiolitis due to human metapneumovirus (J21.1)

H061700 Acute bronchiolitis due to human metapneumovirus

; Acute bronchiolitis due to other specified organisms (J21.8)

H061600 Acute bronchiolitis due to other specified organisms

Hyu1100 [X]Acute bronchiolitis due to other specified organisms

**Genitourinary infections**

; Inflammatory disease of uterus, except cervix (N71)

K41..00 Uterine inflammatory diseases excluding the cervix

K410.00 Acute uterine inflammatory disease

K410z00 Acute uterine inflammatory diseases NOS

K411.00 Chronic uterine inflammatory disease

K411z00 Chronic uterine inflammatory disease NOS

K41z.00 Unspecified uterine inflammatory disease

K41zz00 Unspecified uterine inflammatory disease NOS$

; Inflammatory disease of cervix uteri (N72)

A167000 Tuberculous cervicitis

A981500 Acute gonococcal cervicitis

A983500 Chronic gonococcal cervicitis

K420.00 Cervicitis and endocervicitis

K420000 Cervicitis unspecified

K420200 Acute cervicitis

K420300 Cervicitis with erosion

K420400 Cervicitis with Nabothian cyst

K420500 Cervicitis with ectropion

K420900 Chlamydia cervicitis

; Acute cystitis (N30.0)

K15..00 Cystitis

K150.00 Acute cystitis

; Urinary tract infection, site not specified (N39.0)

K190.00 Urinary tract infection; site not specified

K190500 Urinary tract infection

K190z00 Urinary tract infection; site not specified NOS

; Acute prostatitis (N41.0)

A981200 Acute gonococcal prostatitis

K210.00 Acute prostatitis

**Gastrointestinal infections (A00-A05, A08)**

; Cholera (A00)

A00..00 Cholera

A00z.00 Cholera NOS

Ayu0000 [X]Cholera; unspecified

; Typhoid and paratyphoid fevers (A01)

A01..00 Typhoid and paratyphoid fevers

A010.00 Typhoid fever

A011.00 Paratyphoid fever A

A012.00 Paratyphoid fever B

A013.00 Paratyphoid fever C

A01z.00 Paratyphoid fever NOS

Ayu0100 [X]Paratyphoid fever; unspecified

F007600 Meningitis due to typhoid fever

H24y600 Pneumonia with typhoid fever

; Other salmonella infections (A02)

A02..00 Other salmonella infections

A020.00 Salmonella gastroenteritis

A020.12 Salmonella food poisoning

A021.00 Salmonella septicaemia

A022.00 Localised salmonella infection

A022000 Local salmonella infection unspecified

A022100 Salmonella meningitis

A022200 Salmonella pneumonia

A022300 Salmonella arthritis

A022400 Salmonella osteomyelitis

A022z00 Other local salmonella infection

A023.00 Salmonella sepsis

A02y.00 Other specified salmonella infection

A02z.00 Salmonella infection NOS

Ayu0200 [X]Other specified salmonella infections

Ayu0300 [X]Salmonella infection; unspecified

; Shigellosis (A03)

A03..00 Shigellosis

A03z.00 Shigellosis NOS

A030.00 Shigella dysenteriae (group A)

A031.00 Shigella flexneri (group B)

A032.00 Shigella boydii (group C)

A033.00 Shigella sonnei (group D)

A033.11 Bacillary dysentery Shigella sonnei

A03y.00 Other specified shigella infection

Ayu0400 [X]Other shigellosis

Ayu0500 [X]Shigellosis; unspecified

; Other bacterial intestinal infections (A04)

A070000 Enteropathogenic Escherichia coli infection

A070100 Enterotoxigenic Escherichia coli infection

A070200 Enteroinvasive Escherichia coli infection

A070300 Enterohaemorrhagic Escherichia coli infection

A3B4.00 Escherichia coli infection

A074312 Campylobacter enteritis

A074400 Enteritis due to Yersinia enterocolitica

A3Ay200 Clostridium difficile infection

Ayu0600 [X]Other specified bacterial intestinal infections

Ayu0G00 [X]Other specified intestinal infections

; Other bacterial foodborne intoxications, not elsewhere classified (A05)

A040.00 Staphylococcal food poisoning

A074000 Staphylococcal gastrointestinal tract infection

A041.00 Botulism

A042.00 Clostridium perfringens food poisoning

A3A0211 Clostridium perfringens infection

A044.00 Vibrio parahaemolyticus food poisoning

A04y000 Foodborne Bacillus cereus intoxication

A044.00 Vibrio parahaemolyticus food poisoning

Ayu0800 [X]Other specified bacterial food-borne intoxications

Ayu0900 [X]Bacterial food-borne intoxication; unspecified

;Viral and other specified intestinal infections (A08)

A075.00 Unspecified bacterial enteritis

A076.00 Enteritis due to specified virus

A076000 Enteritis due to adenovirus

A076100 Enteritis due to enterovirus

A076200 Enteritis due to rotavirus

A076300 Enteritis due to norovirus

A076z00 Enteritis due to specified virus NOS

A07y000 Viral gastroenteritis

Ayu0E00 [X]Other viral enteritis

Ayu0F00 [X]Viral intestinal infection; unspecified

Ayu0G00 [X]Other specified intestinal infections

**Septicaemia**

G862.00 Lymphangitis

G862100 Subacute lymphangitis

L452.11 Lymphangitis of breast - obstetric

M03..12 Acute lymphangitis of skin excluding digits of hand or foot

M03zz11 Acute lymphangitis NOS

A021.00 Salmonella septicaemia

A202.00 Septicaemic plague

A223.00 Anthrax septicaemia

A271100 Erysipelothrix septicaemia

A270100 Listeria septicaemia

A362000 Acute meningococcaemia

A362.00 Meningococcal septicaemia

A365.00 Meningococcal meningitis with acute meningococcal septicaem

A366.00 Meningococcal meningitis with meningococcal septicaemia

A545.00 Herpes simplex septicaemia

A98yz12 Gonococcal septicaemia

A98yz11 Gonococcaemia NOS

AB2y300 Candidal septicaemia

L090z00 Septicaemia NOS following abortive pregnancy

L403.00 Puerperal septicaemia

Q407500 Neonatal candida septicaemia

Q40y200 Septicaemia of newborn

R055500 [D]Septic shock

R055511 [D]Septicaemic shock

SP20100 Postoperative septic shock

SP25400 Postoperative septicaemia

SP38000 Septic shock due to transfusion

A38..00 Septicaemia

A380.00 Streptococcal septicaemia

A380000 Septicaemia due to streptococcus, group A

A380100 Septicaemia due to streptococcus, group B

A380300 Septicaemia due to streptococcus pneumoniae

A380400 Septicaemia due to enterococcus

A380500 Vancomycin resistant enterococcal septicaemia

A381.00 Staphylococcal septicaemia

A381000 Septicaemia due to Staphylococcus aureus

A381100 Septicaemia due to coagulase-negative staphylococcus

A382.00 Pneumococcal septicaemia

A383.00 Septicaemia due to anaerobes

A383000 Fusobacterial necrotising tonsillitis??

A383011 Lemierre's syndrome??

A384.00 Septicaemia due to other gram negative organisms

A384000 Gram negative septicaemia NOS

A384100 Haemophilus influenzae septicaemia

A384200 Escherichia coli septicaemia

A384211 E.coli septicaemia

A384300 Pseudomonas septicaemia

A384400 Serratia septicaemia

A38y.00 Other specified septicaemias

A38z.00 Septicaemia NOS

A38z.11 Sepsis

Ayu3E00 [X]Other streptococcal septicaemia

Ayu3F00 [X]Streptococcal septicaemia, unspecified

Ayu3G00 [X]Septicaemia due to other gram-negative organisms

Ayu3H00 [X]Other specified septicaemia

Ayu3J00 [X]Septicaemia, unspecified

H5y0100 Tracheostomy sepsis

L040011 Spontaneous abortion with sepsis

L090y00 Sepsis NOS following abortion/ectopic/molar pregnancy

L40..11 Sepsis - puerperal

Q404y00 Other specified umbilical sepsis

Q404z00 Umbilical sepsis NOS

A272100 Pasteurella septic infection (cat or dogbite)

R106.00 [D]Unspecified bacteraemia

R107.00 [D]Unspecified viraemia

Q40A000 Sepsis of newborn due to Staphylococcusaureus

Q40y011 Congenital sepsis NOS

Table S1. Sensitivity analyses of IRR for acute infections comparing patients with first ever diagnosed depression to patients with no diagnosed depression.

| **Analysis allowing 7 days between recorded infections to count as separate outcome events** | | |
| --- | --- | --- |
|  | **Crude** | **PS-weighted** |
| Any acute infection | 1.17 (1.15 – 1.20) | 1.19 (1.17 – 1.21) |
| Acute respiratory infections | 1.14 (1.12 – 1.17) | 1.16 (1.13 – 1.18) |
| Acute genitourinary infections | 1.22 (1.18 – 1.26) | 1.24 (1.20 – 1.28) |
| Acute gastrointestinal infections | 1.31 (1.18 – 1.45) | 1.31 (1.18 – 1.45) |
| Acute septicaemia | 1.22 (0.99 – 1.50) | 1.23 (1.00 – 1.51) |
| **Analysis allowing 28 days between recorded infections to count as separate outcome events** | | |
| Any acute infection | 1.17 (1.15 – 1.19) | 1.19 (1.17 – 1.21) |
| Acute respiratory infections | 1.14 (1.12 – 1.17) | 1.15 (1.13 – 1.18) |
| Acute genitourinary infections | 1.22 (1.18 – 1.25) | 1.23 (1.20 – 1.27) |
| Acute gastrointestinal infections | 1.31 (1.18 – 1.45) | 1.31 (1.19 – 1.46) |
| Acute septicaemia | 1.20 (0.99 – 1.46) | 1.21 (0.99 – 1.48) |
| **Analysis in patients with no history of cancer within 2 years before cohort entry** | | |
| Any acute infection | 1.17 (1.15 – 1.19) | 1.19 (1.17 – 1.21) |
| Acute respiratory infections | 1.14 (1.12 – 1.17) | 1.15 (1.13 – 1.18) |
| Acute genitourinary infections | 1.22 (1.18 – 1.26) | 1.24 (1.20 – 1.28) |
| Acute gastrointestinal infections | 1.31 (1.18 – 1.45) | 1.31 (1.19 – 1.45) |
| Acute septicaemia | 1.22 (0.98 – 1.51) | 1.23 (1.00 – 1.50) |

Abbreviations: CI, confidence interval; IR, incidence rate; IRR, incidence rate ratios; PS, propensity score;

**Table S2.** Subgroups analysis for the risk of acute respiratory infection comparing patients with first ever diagnosed depression to patients with no diagnosed depression after PS-weighting.

|  | **No. of events** | | **Total person-years of follow-up** | | **Incidence rate per 1000 person-years (95% CI)** | | **Incidence rate ratio (95% CI)** |
| --- | --- | --- | --- | --- | --- | --- | --- |
|  | **No diagnosed depression** | **Diagnosed depression** | **No diagnosed depression** | **Diagnosed depression** | **No diagnosed depression** | **Diagnosed depression** |  |
| **Sex** | | | | | | |  |
| **Men** | 5 967 | 3 763 | 185 514.1 | 106 042.1 | 32.2 (31.4 – 33.0) | 35.5 (34.4 – 36.6) | 1.05 (1.00 – 1.09) |
| **Women** | 19 602 | 14 411 | 302 590.0 | 186 891.9 | 64.8 (63.9 – 65.7) | 77.1 (75.9 – 78.4) | 1.17 (1.14 – 1.20) |
| **GP visits with one year** | | | | | | |  |
| **0 - 2** | 1 803 | 2 480 | 73 510.6 | 76 415.6 | 24.5 (23.4 – 25.7) | 32.5 (31.1 – 33.7) | 1.33 (1.29 – 1.37)* |
| **3 - 5** | 5 276 | 3 478 | 119 087.0 | 63 524.0 | 44.3 (43.1 – 45.5) | 54.8 (52.9 – 56.6) | 1.23 (1.17 – 1.29) |
| **6 - 9** | 6 804 | 4 191 | 121 763.7 | 63 524.0 | 55.9 (54.6 – 57.2) | 66.0 (64.0 – 68.0) | 1.27 (1.22 – 1.33) |
| **10 - 19** | 9 029 | 5 535 | 129 334.4 | 67 177.4 | 69.8 (68.4 – 71.3) | 82.4 (80.2 – 84.6) | 1.19 (1.15 -1.24) |
| **20+** | 3 401 | 2 489 | 43 133.7 | 27 024.7 | 78.8 (76.2 – 81.5) | 92.1 (88.5 – 95.7) | 1.18 (1.11 – 1.26) |
| **Age groups (in years)** | | | | | | |  |
| **18 - 24** | 8 787 | 5 916 | 120 642.7 | 69 197.4 | 72.8 (71.1 – 74.4) | 85.5 (83.3 – 87.7) | 1.16 (1.11 – 1.20) |
| **25 - 34** | 7 386 | 5 287 | 113 789.1 | 67 529.6 | 64.9 (63.4 – 66.4) | 78.3 (76.2 – 80.4) | 1.16 (1.12 – 1.21) |
| **35 - 44** | 4 786 | 3 458 | 97 977.3 | 59 220.5 | 48.9 (47.5 – 50.2) | 58.4 (56.53 – 60.3) | 1.11 (1.06 – 1.17) |
| **45 - 54** | 2 283 | 1 784 | 67 995.7 | 42 077.6 | 33.6 (32.2 – 34.9) | 42.4 (40.4 – 44.4) | 1.17 (1.09 – 1.26) |
| **55 -64** | 1 172 | 905 | 39 712.4 | 24 815.8 | 29.5 (27.8 – 31.2) | 36.5 (34.1 – 38.8) | 1.20 (1.09 – 1.33) |
| **65+** | 999 | 824 | 47 845.3 | 30 087.5 | 20.9 (19.6 – 22.2) | 27.4 (25.5 – 29.3) | 1.31 (1.20 – 1.44)* |

Abbreviations: CI, confidence interval; PS, propensity score; *, POISSON

**Table S3.** Subgroups analysis for the risk of acute genitourinary infection comparing patients with first ever diagnosed depression to patients with no diagnosed depression after PS-weighting.

|  | **No. of Events** | | **Total person-years of follow-up** | | **Incidence rate per 1000 person-years (95% CI)** | | **Incidence rate ratio (95% CI)** |
| --- | --- | --- | --- | --- | --- | --- | --- |
|  | **No diagnosed depression** | **Diagnosed depression** | **No diagnosed depression** | **Diagnosed depression** | **No diagnosed depression** | **Diagnosed depression** |  |
| **Sex** | | | | | | |  |
| **Men** | 1 543 | 1 230 | 185 514.1 | 106 042.1 | 8.3 (7.9 – 8.7) | 11.6 (11.0 – 12.3) | 1.32 (1.20 – 1.46) |
| **Women** | 12 970 | 9 776 | 302 590.0 | 186 891.9 | 42.9 (42.1 – 43.6) | 52.3 (51.3 – 53.4) | 1.20 (1.17 – 1.24) |
| **GP visits with one year** | | | | | | |  |
| **0 - 2** | 703 | 1 044 | 73 510.6 | 76 415.6 | 9.6 (8.9 – 10.3) | 13.7 (12.8 – 14.5) | 1.26 (1.13 – 1.40) |
| **3 - 5** | 2 450 | 1 780 | 119 087.0 | 63 524.0 | 20.6 (19.8 – 21.4) | 28.0 (26.7 – 29.3) | 1.37 (1.27 – 1.47) |
| **6 - 9** | 3 814 | 2 161 | 121 763.7 | 63 524.0 | 31.3 (30.3 – 32.3) | 36.6 (35.1 – 38.2) | 1.22 (1.14 – 1.30) |
| **10 - 19** | 5 636 | 3 673 | 129 334.4 | 67 177.4 | 43.6 (42.4 – 44.7) | 54.7 (52.9 – 56.4) | 1.32 (1.25 – 1.39) |
| **20+** | 2 924 | 2 345 | 43 133.7 | 27 024.7 | 67.8 (65.3 – 70.2) | 86.8 (83.3 – 90.3) | 1.31 (1.22 – 1.41) |
| **Age groups (in years)** | | | | | | |  |
| **18 - 24** | 4 168 | 2 830 | 120 642.7 | 69 197.4 | 34.6 (33.5 – 36.6) | 40.9 (39.4 – 42.4) | 1.16 (1.10 – 1.23) |
| **25 - 34** | 2 820 | 2 065 | 113 789.1 | 67 529.6 | 24.8 (23.9 – 25.7) | 30.6 (29.3 – 31.9) | 1.18 (1.10 – 1.26) |
| **35 - 44** | 2 192 | 1 495 | 97 977.3 | 59 220.5 | 22.4 (21.4 – 23.3) | 25.2 (24.0 – 26.5) | 1.11 (1.06 – 1.17) |
| **45 - 54** | 1 464 | 974 | 67 995.7 | 42 077.6 | 21.5 (20.4 – 22.6) | 23.2 (21.7 – 24.6) | 1.04 (0.94 – 1.15) |
| **55 -64** | 1077 | 810 | 39 712.4 | 24 815.8 | 27.1 (25.5 – 28.7) | 32.6 (30.4 – 34.9) | 1.19 (1.06 – 1.34) |
| **65+** | 2 835 | 2 837 | 47 845.3 | 30 087.5 | 59.3 (57.1 – 61.44) | 94.3 (90.8 – 97.8) | 1.69 (1.57 – 1.82) |

Abbreviations: CI, confidence interval; PS, propensity score; *, POISSON

**Table S4.** Subgroups analysis for the risk of acute gastrointestinal infection comparing patients with first ever diagnosed depression to patients with no diagnosed depression after PS-weighting.

|  | **No. of Events** | | **Total person-years of follow-up** | | **Incidence rate per 1000 person-years (95% CI)** | | **Incidence rate ratio (95% CI)** |
| --- | --- | --- | --- | --- | --- | --- | --- |
|  | **No diagnosed depression** | **Diagnosed depression** | **No diagnosed depression** | **Diagnosed depression** | **No diagnosed depression** | **Diagnosed depression** |  |
| **Sex** | | | | | | |  |
| **Men** | 283 | 206 | 185 514.1 | 106 042.1 | 1.5 (1.4 – 1.7) | 1.9 (1.7 – 2.2) | 1.1.14 (0.96 – 1.37) |
| **Women** | 537 | 475 | 302 590.0 | 186 891.9 | 1.8 (1.6 – 1.9) | 2.5 (2.3 – 2.8) | 1.40 (1.24 – 1.59) |
| **GP visits with one year** | | | | | | |  |
| **0 - 2** | 68 | 89 | 73 510.6 | 76 415.6 | 0.9 (0.7 – 1.2) | 1.2 (0.9– 1.4) | 1.18 (0.87 – 1.62) |
| **3 - 5** | 159 | 146 | 119 087.0 | 63 524.0 | 1.3 (1.1 – 1.5) | 2.3 (1.9 – 2.7) | 1.66 (1.32 – 2.08) |
| **6 - 9** | 208 | 133 | 121 763.7 | 63 524.0 | 1.7 (1.5 – 1.9) | 2.3(1. 9 – 2.6) | 1.32 (0.83 – 2.08) |
| **10 - 19** | 267 | 188 | 129 334.4 | 67 177.4 | 2.1 (1.8 – 2.3) | 2.8 (2.4 – 3.2) | 1.36 (1.13 – 1.64) |
| **20+** | 147 | 125 | 43 133.7 | 27 024.7 | 3.4 (2.9 – 4.0) | 4.6 (3.8 – 5.4) | 1.33 (1.04 – 1.69) |
| **Age groups (in years)** | | | | | | |  |
| **18 - 24** | 304 | 231 | 120 642.7 | 69 197.4 | 2.5 (2.2 – 2.8) | 3.3 (2.9 – 3.8) | 1.34 (1.12 – 1.59) |
| **25 - 34** | 199 | 167 | 113 789.1 | 67 529.6 | 1.8 (1.5 – 2.0) | 25 (2.1 – 2.9) | 1.30 (1.06 – 1.59) |
| **35 - 44** | 107 | 93 | 97 977.3 | 59 220.5 | 1.1 (0.9 – 1.3) | 1.6 (1.3 – 1.9) | 1.06 (0.98 – 1.14) |
| **45 - 54** | 80 | 55 | 67 995.7 | 42 077.6 | 1.2 (0.9 – 1.4) | 1.3 (1.0 – 1.7) | 1.02 (0.71 – 1.44) |
| **55 -64** | 47 | 42 | 39 712.4 | 24 815.8 | 1.2 (0.9 – 1.5) | 1.6 (1.2 – 2.1) | 1.36* (1.27 – 1.45) |
| **65+** | 92 | 93 | 47 845.3 | 30 087.5 | 1.9 (1.5 – 2.3) | 3.1 (25 – 3.7) | 1.63 (1.20 – 2.22) |

Abbreviations: CI, confidence interval; PS, propensity score; *, POISSON

**Table S5.** Subgroups analysis for the risk of acute septicaemia comparing patients with diagnosed first ever depression to patients with no diagnosed depression after PS-weighting.

|  | **No. of Events** | | **Total person-years of follow-up** | | **Incidence rate per 1000 person-years (95% CI)** | | **Incidence rate ratio (95% CI)** |
| --- | --- | --- | --- | --- | --- | --- | --- |
|  | **No diagnosed depression** | **Diagnosed depression** | **No diagnosed depression** | **Diagnosed depression** | **No diagnosed depression** | **Diagnosed depression** |  |
| **Sex** | | | | | | |  |
| **Men** | 92 | 76 | 185 514.1 | 106 042.1 | 0.5 (0.4 – 0.6) | 0.7 (0.6 – 0.9) | 1.31 (0.95 – 179) |
| **Women** | 132 | 99 | 302 590.0 | 186 891.9 | 0.4 (0.4 – 0.5) | 0.5 (0.4 – 0.6) | 1.18 (0.90 – 1.54) |
| **GP visits with one year** | | | | | | |  |
| **0 - 2** | 8 | 23 | 73 510.6 | 76 415.6 | 0.1 (0.1 – 1.2) | 0.3 (0.2 – 0.4) | 2.49 (1.11 – 5.58) |
| **3 - 5** | 35 | 23 | 119 087.0 | 63 524.0 | 0.3 (0.2 – 0.4) | 0.4 (0.2 – 0.5) | 175 (0.98 – 3.12) |
| **6 - 9** | 47 | 30 | 121 763.7 | 63 524.0 | 0.4 (0.3 – 0.5) | 0.5 (0.3 – 0.7) | 1.32 (0.83 – 2.08) |
| **10 - 19** | 73 | 51 | 129 334.4 | 67 177.4 | 0.6 (0.4 – 0.7) | 0.8 (0.6 – 1.0) | 1.43(0.97 – 2.11 |
| **20+** | 73 | 48 | 43 133.7 | 27 024.7 | 1.7 (1.3 – 2.1) | 1.8 (1.3 – 2.3) | 1.01 (0.68 – 1.49 |
| **Age groups (in years)** | | | | | | |  |
| **18 - 24** | 37 | 25 | 120 642.7 | 69 197.4 | 0.3 (0.2 – 0.4) | 0.4 (0.2 – 0.5) | 1.27 (0.75 – 2.16) |
| **25 - 34** | 35 | 32 | 113 789.1 | 67 529.6 | 0.3 (0.2 – 0.4) | 0.5 (0.3 – 0.6) | 1.42 (0.88 – 2.30) |
| **35 - 44** | 26 | 22 | 97 977.3 | 59 220.5 | 0.3 (0.2 – 0.4) | 0.4 (0.2 – 0.5) | 1.32 (1.00 – 1.75) |
| **45 - 54** | 25 | 15 | 67 995.7 | 42 077.6 | 0.4 (0.2 – 0.5) | 0.4 (0.2 – 0.5) | 0.73 (0.38 – 1.38) |
| **55 -64** | 24 | 15 | 39 712.4 | 24 815.8 | 0.6 (0.4 – 0.8) | 0.6 (0.3 – 0.9) | 0.95 (048 – 1.87) |
| **65+** | 87 | 66 | 47 845.3 | 30 087.5 | 1.8(1.4 – 2.2) | 2.2 (1.7 – 2.7) | 1.37 (0.95 – 1.99) |

Abbreviations: CI, confidence interval; PS, propensity score; *, POISSON

**Table S6**. Baseline characteristics of patients with first ever diagnosed depression and patients with no diagnosed depression with or without a hospital-based diagnosis of infection recorded in HES data.

|  | **Without diagnosed depression** | | **Diagnosed depression** | |
| --- | --- | --- | --- | --- |
|  | **With infection recorded in HES data** | **Without infection recorded in HES data** | **With infection recorded in HES data** | **Without infection recorded in HES data** |
| **Total no. of patients** | 285 923 | | 285 923 | |
| **No. of patients with HES linkage** | 146515 (51.24) | | 146049 (51.05) | |
| **No. of patients** | 182 (0.1) | 146333 (99.9) | 2062 (1.4) | 143987 (98.6) |
| **No. of GP visits (365 d prior), mean (SD)** | 14.5 (10.9) | 9.6 (8.5) | 16.2 (13.5) | 8.3 (8.3) |
| **Age, mean (SD), y** | 41.1 (20.2) | 38.9 (17.4) | 56.2 (25.7) | 39 (17.3) |
| **Sex** | | | | |
| **Male** | 45 (24.7) | 54983 (37.6) | 814 (38.1) | 54417 (37.8) |
| **Female** | 137 (75.3) | 91350 (62.4) | 1324 (61.9) | 89494 (62.2) |
| **BMI** |  |  |  |  |
| 12.0-18.4 | 3 (1.6) | 3762 (2.6) | 90 (4.2) | 4007 (2.8) |
| 18.5-24.9 | 66 (36.3) | 52157 (35.6) | 704 (32.9) | 49342 (34.3) |
| 25.0 - 29.9 | 41 (22.5) | 34456 (23.5) | 505 (23.6) | 31660 (22) |
| 30.0 - 60.0 | 41 (22.5) | 23097 (15.8) | 393 (18.4) | 23048 (16) |
| **Unknown** | 31 (17) | 32861 (22.5) | 446 (20.9) | 35854 (24.9) |
| **Alcohol status** | | | | |
| Low risk consumption (<= 14 units) | 136 (74.7) | 100242 (68.5) | 1551 (72.5) | 96017 (66.7) |
| High risk consumption (14+ units) | 10 (5.5) | 8863 (6.1) | 131 (6.1) | 9175 (6.4) |
| Unknown | 36 (19.8) | 37228 (25.4) | 456 (21.3) | 38719 (26.9) |
| **Smoking status** | | | | |
| Non-smoker | 101 (55.5) | 77536 (53) | 795 (37.2) | 60619 (42.1) |
| Current smoker | 47 (25.8) | 27279 (18.6) | 493 (23.1) | 38695 (26.9) |
| Past smoker | 27 (14.8) | 29609 (20.2) | 683 (31.9) | 28845 (20) |
| Unknown | 7 (3.8) | 11909 (8.1) | 167 (7.8) | 15752 (10.9) |
| **Comorbidities** | | | | |
| Asthma | 36 (19.8) | 28811 (19.7) | 495 (23.2) | 26067 (18.1) |
| COPD | 5 (2.7) | 1656 (1.1) | 201 (9.4) | 1744 (1.2) |
| Arrhythmia | 2 (1.1) | 2580 (1.8) | 97 (4.5) | 2375 (1.7) |
| Congestive heart failure | 6 (3.3) | 1402 (1) | 178 (8.3) | 1313 (0.9) |
| Ischemic heart disease | 7 (3.8) | 4427 (3) | 287 (13.4) | 4301 (3) |
| Myocardial infarction | 1 (0.5) | 2065 (1.4) | 156 (7.3) | 1847 (1.3) |
| Hyperlipidaemia | 16 (8.8) | 7788 (5.3) | 222 (10.4) | 6283 (4.4) |
| Diabetes mellitus | 25 (13.7) | 8873 (6.1) | 299 (14) | 5938 (4.1) |
| Ulcer | 3 (1.6) | 1997 (1.4) | 92 (4.3) | 2176 (1.5) |
| Pneumonia | 5 (2.7) | 2898 (2) | 101 (4.7) | 3011 (2.1) |
| Diagnosed renal diseases | 10 (5.5) | 3211 (2.2) | 135 (6.3) | 2923 (2) |
| Hypothyroidism | 12 (6.6) | 6571 (4.5) | 149 (7) | 4614 (3.2) |
| Cancer | 13 (7.1) | 4215 (2.9) | 179 (8.4) | 3740 (2.6) |
| **Comedication (180 d prior)** | | | | |
| Opioid | 13 (7.1) | 6493 (4.4) | 246 (11.5) | 6494 (4.5) |
| Antipsychotics | 3 (1.6) | 2992 (2) | 104 (4.9) | 3034 (2.1) |
| Agents acting on RAS | 31 (17) | 13887 (9.5) | 511 (23.9) | 9711 (6.7) |
| Beta-blocker | 10 (5.5) | 9333 (6.4) | 271 (12.7) | 7587 (5.3) |
| Calcium channel blockers | 20 (11) | 8018 (5.5) | 292 (13.7) | 5793 (4) |
| Thrombocyte aggregation inhibitors | 22 (12.1) | 8399 (5.7) | 475 (22.2) | 7234 (5) |
| Anticoagulants | 6 (3.3) | 2180 (1.5) | 114 (5.3) | 1286 (0.9) |
| Statins | 24 (13.2) | 11768 (8) | 428 (20) | 8797 (6.1) |
| Diuretics | 29 (15.9) | 11202 (7.7) | 557 (26.1) | 8396 (5.8) |
| Proton pump inhibitors | 25 (13.7) | 10891 (7.4) | 438 (20.5) | 9169 (6.4) |
| Systemic corticosteroids | 10 (5.5) | 6144 (4.2) | 240 (11.2) | 4365 (3) |

Abbreviations: BMI, body mass index; CED, Cohort entry date; COPD, chronic obstructive pulmonary disease; DVT, deep vein thrombosis, GP, general practitioner; PE, pulmonary embolism, RAS, renin-angiotensin- system; SD, standard deviation; Std Diff, absolute standardised difference;
